# Mental health and binge-watching among Brazilian and Canadian university students: a study using latent class analysis and an emotional vulnerability model

**DOI:** 10.1192/j.eurpsy.2026.10846

**Published:** 2026-07-31

**Authors:** A. L. Monezi Andrade, G. A. Oliveira, L. S. da Silva, L. B. Nucci, H. S. Kim, W. A. de Oliveira

**Affiliations:** 1 School of Life Sciences, Pontifical Catholic University of Campinas, Campinas, Brazil; 2 Departament of Psychology, Toronto Metropolitan University, Toronto, Canada

## Abstract

**Introduction:**

Binge-watching (BW) has become a common behavior among university students and is linked to negative effects on mental health. A lot of evidence indicates that problematic BW may be connected to emotional symptoms, lower quality of life, and higher risks of emotional vulnerability.

**Objectives:**

This study examined patterns of BW in a multicenter Brazil-Canada sample using Latent Class Analysis (LCA) and explored their relationships with emotional vulnerability (depression, anxiety, stress, and emotional dysregulation) and quality of life.

**Methods:**

This multicenter cross-sectional study involved 1,417 university students (Brazil, n = 1,191; Canada, n = 206). Participants completed a sociodemographic questionnaire, the BW scales, the DASS-21, the DERS-18, and the WHOQOL-Bref. The LCA was performed based on problematic BW involvement items. The resulting classes were compared in terms of emotional symptoms, emotional dysregulation, and quality of life using chi-square and ANOVA. The study received approval from ethics committees in both Brazil and Canada.

**Results:**

The LCA identified three classes with increasing levels of BW involvement. The classes with higher involvement exhibited significantly higher scores for depression, anxiety, and stress, along with greater emotional dysregulation and poorer quality of life compared to the classes with lower involvement. This pattern was consistent across both countries, supporting the cross-cultural robustness of the findings.

**Conclusions:**

We found that problematic BW patterns are linked to higher emotional vulnerability and poorer quality of life in university students in Brazil and Canada.

**Disclosure of Interest:**

None Declared

